# Bilateral thoracic paravertebral block combined with general anesthesia vs. general anesthesia for patients undergoing off-pump coronary artery bypass grafting: a feasibility study

**DOI:** 10.1186/s12871-019-0768-9

**Published:** 2019-06-12

**Authors:** Lixin Sun, Qiujie Li, Qiang Wang, Fuguo Ma, Wei Han, Mingshan Wang

**Affiliations:** 1 Department of Anesthesiology, Qingdao Municipal Hospital, Qingdao, 266011 Shandong China; 2 Department of Respiratory Medicine, Qingdao Municipal Hospital, 1 Jiaozhou Road, Qingdao, 266011 Shandong China

**Keywords:** Nerve block, Thoracic vertebra, Anesthesia, General

## Abstract

**Background:**

Whether thoracic paravertebral block (PVB) is useful in patients undergoing off-pump coronary artery bypass grafting (OPCABG) remains unknown. This study aimed to investigate the feasibility of bilateral PVB combined with general anesthesia (GA) in patients undergoing OPCABG.

**Methods:**

This feasibility study assessed 60 patients scheduled for OPCABG at the Qingdao Municipal Hospital in 2016–2017. Patients were randomly assigned to receive nerve stimulator-guided bilateral PVB combined with GA (PVB + GA) or GA alone (*n* = 30/group). Patients were asked to rate rest and cough pain hourly after the surgery. The primary endpoint was the visual analogue scale (VAS) pain score within 48 h postoperatively. Secondary endpoints were rescue analgesia and morphine consumption, fentanyl dose within 48 h postoperatively, as well as operative time, time to extubation, intensive care unit (ICU) stay, hospital stay and other postoperative adverse events.

**Results:**

Both rest and cough pains were lower in the PVB + GA group at 12, 24, 36, and 48 h after surgery compared with the GA group. There were fewer patients who needed rescue analgesia in the PVB + GA group at 12 and 24 h than in the GA group. Morphine consumptions at 24 and 48 h were lower in the PVB + GA group compared with the GA group. Time to extubation (*P* = 0.035) and ICU stay (*P* = 0.028) were shorter in the PVB + GA group compared with the GA group. AEs showed no differences between the two groups.

**Conclusions:**

Nerve stimulator-guided bilateral thoracic PVB combined with GA in OPCABG is associated with a reduced rescue analgesia and morphine consumption, compared to GA.

## Background

Off-pump coronary artery bypass grafting (OPCABG) is a type of bypass surgery performed on beating heart, without cardiopulmonary bypass (CPB). OPCABG has been developed in Russia mainly to avoid the complications of CPB [1]. The popularity of OPCABG has been declining over the past years in developed countries, but the rate of OPCABG is currently increasing in some countries such as China and India [2]. Nevertheless, the benefits of OPCABG are debatable [3] and it could benefit only some selected patients [4–6].

Thoracic epidural anesthesia (TEA) has been successfully applied in heart surgery and confirmed to have a myocardial protective effect [7–10]. Nevertheless, a cardiac surgery is usually performed in patients receiving anticoagulant therapy and may be associated with an increased risk of an epidural hematoma. The incidence of epidural hematoma has been estimated to be between 1:150,000 and 1:1528 [11]. Furthermore, TEA may also be complicated by hypotension, urinary retention and pulmonary complications related to respiratory muscle blockade in some patients [12, 13]. Although a clinical study [14] has revealed important benefits for TEA in cardiac surgery, its use is still debatable because of the potential risks.

Recently, there has been increasing interest in alternative regional techniques, particularly thoracic paravertebral block (PVB), which offers optimal pain control with a better side effects profile [15, 16]. Compared with TEA, PVB can provide comparable pain relief, fewer complications, faster recovery, shorter hospitalization, and lower incidence of postoperative chronic pain [17, 18].

The safety and efficacy of segmental PVB has been reported for postoperative analgesia after modified minimally invasive Heart-Port access cardiac surgery [19], but whether bilateral thoracic paravertebral block can be safely and effectively used in OPCABG remains unknown. We hypothesized that PVB could be useful in patients undergoing OPCABG. Therefore, this study aimed to investigate the feasibility of bilateral PVB combined with general anesthesia (GA) in patients undergoing OPCABG, assessing pain (visual analogue scale [VAS] as primary endpoint and rescue analgesia and morphine consumption within 48 h postoperatively, operative time, dose of fentanyl, time to extubation, intensive care unit (ICU) stay, hospital stay, intraoperative parameters (e.g. bradycardia, tachycardia, hypotension and hypertension) and postoperative adverse events (AEs) as secondary endpoints. This was a pilot study comparing PVB combined with GA vs. GA alone in order to observe the advantages and disadvantages of PVB.

## Methods

### Patients and study design

This was a feasibility study of patients scheduled to undergo OPCABG at the Qingdao Municipal Hospital between July 2016 and May 2017. All patients received preoperative physical examination and plain X-ray. The inclusion criteria were: 1) planned to undergo OPCABG; 2) 50–75 years old; 3) body mass index (BMI) < 30 kg/m^2^; 4) ASA II or III; and 5) elective surgery. The exclusion criteria were: 1) spine malformation; 2) vertebral space-occupying lesion; 3) infection at the site of paravertebral injection; 4) left ventricular ejection fraction (LVEF) < 40%; 5) endocrine disease; 6) metabolic disease; 7) extracorporeal circulation; 8) allergies; 9) severe hepatic (alanine transaminase [ALT] or aspartate transaminase [AST] > 3 times the upper limit of normal) or renal dysfunction (serum creatinine [SCr] > 178 mmol/L and blood urea nitrogen [BUN] > 9 mmol/L); 10) valvular disease; 11) intra-aortic balloon pump; or 12) neurologic or psychotic disorders. The study was approved by the ethics committee of Qingdao Municipal Hospital (No. 20140806–1). Each patient provided a written informed consent.

### Randomization and blinding

The patients were randomized using sequential sealed envelopes prepared by an independent statistician using a computer-generated random number table. Patients were randomly divided into two groups: the bilateral thoracic PVB combined with GA group (PVB + GA group), and the GA group (GA group). The postoperative assessors were blinded to grouping.

### Anesthesia

All patients received their usual medication on the day of operation, followed by premedication with intramuscular morphine 0.1 mg/kg and midazolam 0.05 mg/kg. Upon arrival in the operating room, 100% oxygen was administered, and peripheral vein Ringer lactate solution was infused at 6–8 ml/kg/h.

The patients were monitored with radial artery pressure, heart rate (HR), electrocardiogram (ECG), oxygen saturation (SpO_2_), end-tidal carbon dioxide (ETCO_2_), and other hemodynamic parameters using a Datex multi-parameter monitor (GE Healthcare, Waukesha, WI, USA). The flow-directed pulmonary artery catheter and central venous catheter (Arrow International Inc., Asheboro, NC, USA) were placed through the right internal jugular vein in both groups.

In the PVB + GA group, bilateral thoracic PVB was performed according to the nerve stimulator-guided technique [20–23] combined with the loss of resistance technique for proper location of the paravertebral space (PVS). Briefly, patients in the right lateral decubitus position received intradermal lidocaine (1%) at T_3–4_ PVS. An insulated needle attached to a nerve stimulator was advanced between the transverse process of the third and fourth vertebrae, and the current intensity of the nerve stimulator was set to 2–5 mA during initial simulation, and subsequently reduced to 0.4–0.8 mA. PVS could also be identified with the “loss-of-resistance” technique to ensure technical success [23, 24], but neuromuscular stimulation was the primary criterion in cases in which loss of resistance could not be felt. After ensuring the absence of blood, air, or cerebrospinal fluid, a 20G catheter was passed through the needle, with 3 cm of the catheter left in the PVS.

After the catheters had been secured, the patient was turned onto the supine position. 0.375% of ropivacaine 20 ml were injected by 2 time with 5 min interval (5 ml in the first time and 15 ml in the second time), followed by 0.375% of ropivacaine infused at 5 ml/h until 30 min before the end of operation [25]. Analgesia block levels were tested by the pinprick method at the middle of the chest. If analgesia block level was less than 2 dermatomes, the patient was withdrawn. Bilateral thoracic PVB was not performed in the GA group.

All patients in both groups received GA. GA was induced by midazolam 0.05 mg/kg, etomidate 0.3 mg/kg, fentanyl 4 μg/kg, and vecuronium 0.1 mg/kg. After endotracheal intubation, patients were mechanically ventilated to maintain ETCO_2_ between 35 and 40 mmHg. The nasopharyngeal temperature and urine volume were monitored. Warming blankets were used to maintain the nasopharyngeal temperature at 36.5–37.5 °C. Anesthesia was maintained by sevoflurane 1 MAC. Fentanyl 10–20 μg/kg and vecuronium 0.1 mg/kg were given when indicated. At the end of the operation, the catheter was removed in the PVB + GA group in order to observe the effect of PVB on postoperative morphine use. All patients in both groups were transferred to the intensive care unit (ICU) without extubation. Beginning at the end of the operation, all patients in both groups received patient-controlled analgesia (PCA) using morphine (1 mg/ml) for 48 h at a loading dose of 2 mg, continuous infusion dose of 0.5 mg/h, bolus of 1 mg, locking time of 10 min, and maximum dose of 20 mg/4 h.

### Assessments

Using the visual analogue scale (VAS; 0 mm = no pain, 100 mm = worst pain imaginable), the patients were asked to rate their pain at rest and during coughing every hour after their arrival and return to consciousness in ICU. If the VAS score was > 5 at rest, rescue analgesia was given with morphine 5 mg IV.

Intraoperative adverse events (AEs), including bradycardia, tachycardia, hypotension, hypertension, and postoperative AEs were recorded. Bradycardia was defined as a heart rate < 50 bpm and treated with intravenous atropine 0.01 mg/kg. Tachycardia was defined as a heart rate > 90 bpm and treated with intravenous esmolol 20 mg. Hypotension was defined as a 20% decrease in systolic blood pressure (SBP) from baseline and treated with intravenous noradrenaline 5 μg. Hypertension was defined as a 20% increase in SBP from baseline and treated with intravenous urapidil hydrochloride 10 mg. All hemodynamic drugs were repeated as required.

### Endpoints

The primary endpoint was the pain scores within 48 h postoperatively. The secondary endpoints were the rescue analgesia and morphine consumption, dose of fentanyl within 48 h postoperatively, as well as operative time, time to extubation (defined as the time from the end of surgery to the extubation), ICU stay, hospital stay and postoperative AEs (including bradycardia, tachycardia, hypotension and hypertension).

### Statistical analysis

Since this was a pilot study, the sample size was estimated by referring to similar studies [26] rather than an accurate calculation. Continuous data (age, BMI, morphine consumption, operative time, dose of fentanyl, time to extubation, ICU stay, and hospital stay) were expressed as mean ± standard deviation (SD) and analyzed using Student t test for intergroup comparisons. Categorical data (gender, ASA score, diabetes mellitus, chronic obstructive pulmonary disease, renal dysfunction, hypertension, rescue analgesia, and intraoperative and postoperative AEs) were expressed as frequency (percentage) and analyzed using the chi-square test. Ranked data (rest pain score and cough pain score) were expressed as median [IQR] and analyzed using the Wilcoxon rank sum test. All variables, except for pain scores, were baseline characteristics or secondary endpoints. Statistical analysis was performed using PASW Statistics 18.0 (SPSS Inc., Chicago, NY, USA). Two-sided *P*-values < 0.05 were considered statistically significant.

## Results

### Patients

**Fig. 1:**
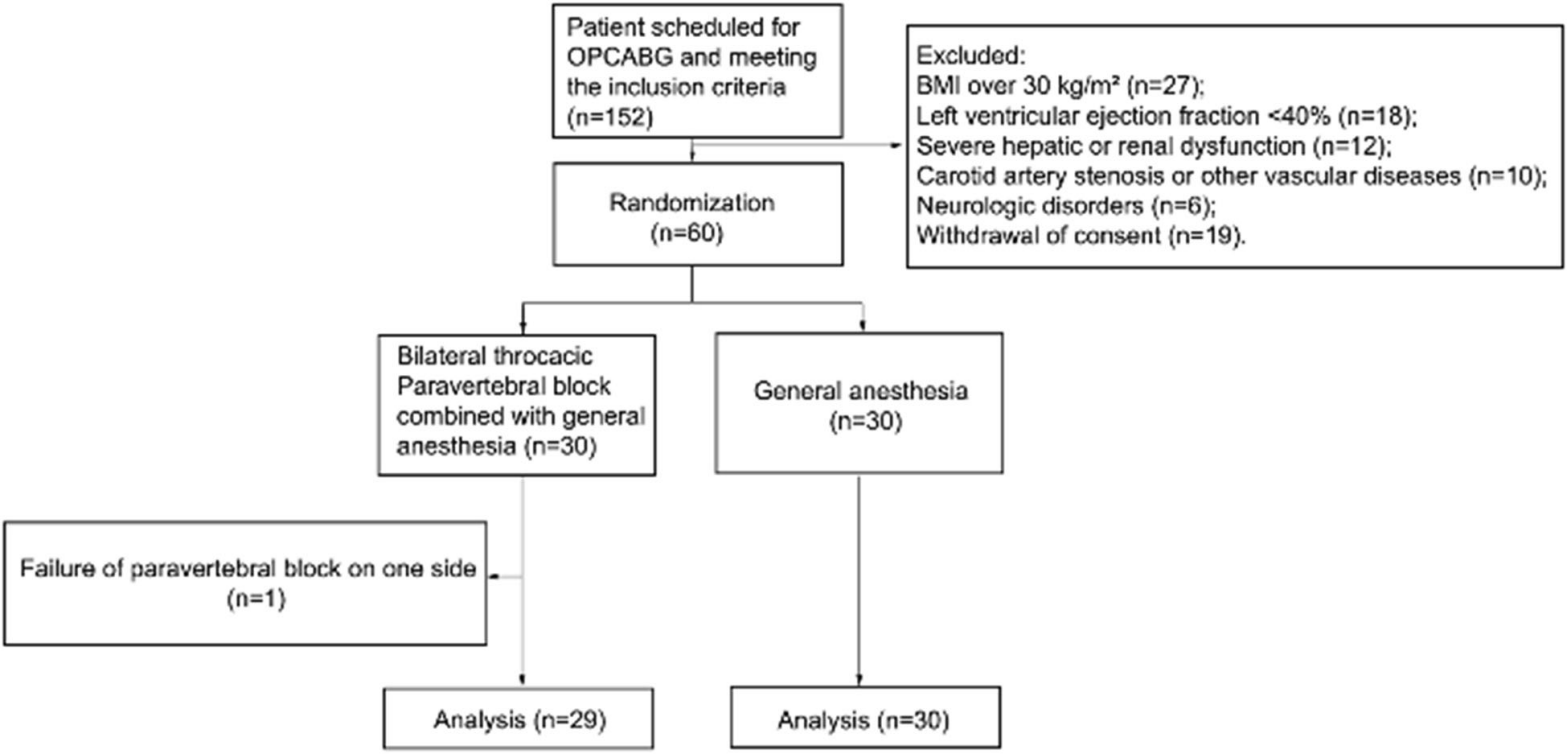
Study flowchart. BMI, body mass index; OPCABG, off-pump coronary artery bypass grafting

Figure 1 presents the patient flowchart. One hundred and fifty-two patients met the inclusion criteria and 92 of them were excluded from the study, including 27 patients due to BMI over 30 kg/m^2^, 18 patients due to LVEF < 40%, 12 patients due to severe hepatic or renal dysfunction, 10 patients due to carotid artery stenosis or other vascular diseases, 6 patients due to neurologic disorders and 19 patients due to withdrawal of consent. Sixty patients underwent randomization, with 30 patients in each group. One patient was withdrawn from the study due to the failure of PVB on one side and received GA alone. Table 1 presents the baseline characteristics of the patients.

**Table 1:** Baseline characteristics of the patients

Characteristics	PVB + GA (*n* = 29)	GA (*n* = 30)
Male, n (%)	23 (79.3)	21 (70.0)
Age (years)	68.2 ± 10.5	70.6 ± 11.7
BMI (kg/m^2^)	23.0 ± 6.6	24.2 ± 5.1
ASA (II/III)	7/10	9/9
LVEF (%)	55.8 ± 8.1	56.6 ± 10.7
DM, n (%)	13 (44.8)	15 (50.0)
COPD, n (%)	3 (10.3)	5 (16.7)
Renal dysfunction, n (%)	3 (10.3)	2 (6.7)
Hypertension, n (%)	20 (69.0)	18 (60.0)

*PVB* Paravertebral block, *GA* General anesthesia, *BMI* Body mass index, *ASA* American Standards Association, *LVEF* Left ventricular ejection fraction, *DM* Diabetes mellitus, *COPD* Chronic obstructive pulmonary disease. There were no significant differences between the two groups

### Postoperative pain

Both rest and cough pain scores were lower in the PVB + GA group at 12, 24, 36, and 48 h after surgery compared with the GA group (rest pain: 12 h: 3 [2, 3] vs. 3 [3, 3], *P* = 0.004; 24 h: 3 [2, 3] vs. 3 [3, 4], *P* = 0.007; 36 h: 3 [2, 3] vs. 3 [2, 4], *P* = 0.018; 48 h: 2 [2, 3] vs. 3 [3, 4], *P* = 0.010; cough pain: 12 h: 4 [3, 4] vs. 4 [4, 5], P = 0.007; 24 h: 3 [3, 5] vs. 4 [4, 5], *P* = 0.017; 36 h: 4 [3, 5] vs. 5 [3, 6], *P* = 0.048; 48 h: 4 [3, 4] vs. 5 [3, 6], *P* = 0.023). There were fewer patients who received rescue analgesia at 12 and 24 h in the PVB + GA group than in the GA group (12 h: 0% vs. 16.7%, *P* = 0.034; 24 h: 3.4% vs. 20.0%, *P* = 0.049). The number of patients who received rescue analgesia at 36 and 48 h in the two groups were similar. Morphine consumption at 24 and 48 h was lower in the PVB + GA group compared with the GA group (24 h: 25.6 ± 7.3 vs. 30.7 ± 9.0 mg, *P* = 0.033; 48 h: 47.6 ± 13.5 vs. 54.3 ± 16.1 mg, *P* = 0.041) (Table 2).

**Table 2:** Comparison of VAS scores, rescue analgesia and morphine consumption between groups

Variable	Group	n	PCA 12 h	PCA 24 h	PCA 36 h	PCA 48 h
Rest pain	PVB + GA	29	3 [2, 3]	3 [2, 3]	3 [2, 3]	2 [2, 3]
	GA	30	3 [3, 3]	3 [3, 4]	3 [2, 4]	3 [3, 4]
	P		0.015	0.023	0.026	0.042
Cough pain	PVB + GA	29	3 [2, 3]	3 [2, 3]	3 [2, 3]	2 [2, 3]
	GA	30	3 [3, 3]	3 [3, 4]	3 [2, 4]	3 [3, 4]
	P		0.023	0.030	0.027	0.034
Rescue analgesia	PVB + GA	29	0 (0)	1 (3.4)	3 (10.3)	2 (6.9)
	GA	30	5 (16.7)	6 (20.0)	6 (20.0)	7 (23.3)
	P		0.034	0.049	0.302	0.079
Morphine consumption	PVB + GA	29	–	25.6 ± 7.3	–	47.6 ± 13.5
	GA	30	–	30.7 ± 9.0	–	54.3 ± 16.1
	P			0.033		0.041

*VAS* Visual analogue scale, *PCA* Patient-controlled analgesia, *PVB* Paravertebral block, *GA* General anesthesia

### Clinical characteristics

Table 3 shows that the time to extubation was shorter in the PVB + GA group compared with the GA group (5.8 ± 1.5 vs. 7.3 ± 1.7 h, *P* = 0.035), as well as the ICU stay (16.3 ± 3.7 vs. 20.2 ± 4.1 h, *P* = 0.028). The dose of fentanyl was lower in the PVB + GA group compared with the GA group (1.2 ± 0.2 vs. 1.5 ± 0.3 mg, *P* = 0.022). There were no differences in operative time and hospital stay between the two groups.

**Table 3:** Clinical characteristics of the patients

Variable	PVB + GA (n = 29)	GA (n = 30)	*P* ^*^
Operative time (min)	168 ± 23	176 ± 28	0.279
Dose of fentanyl (mg)	1.2 ± 0.2	1.5 ± 0.3	0.022
Time to extubation (h)	5.8 ± 1.5	7.3 ± 1.7	0.035
ICU stay (h)	16.3 ± 3.7	20.2 ± 4.1	0.028
Hospital stay (d)	9.6 ± 2.1	10.1 ± 2.3	0.459

*PVB* Paravertebral block, *GA* General anesthesia, *ICU* Intensive care unit. *All variables were analyzed using Student t test

### Adverse events

Table 4 presents the intraoperative and postoperative AEs in the two groups. The occurrences of tachycardia (3.4% vs. 27.8%, P = 0.035) and hypertension (0% vs. 16.7%, *P* = 0.008) were lower in the PVB + GA group compared with the GA group. There were no differences in the occurrences of bradycardia, hypotension, and postoperative AEs between the two groups.

**Table 4:** Intraoperative and postoperative AEs

Variable, n (%)	PVB + GA (n = 29)	GA (n = 30)	*P* ^*^
Intraoperative AE			
Bradycardia	5 (17.2)	3 (10.0)	0.334
Tachycardia	1 (3.4)	5 (27.8)	0.035
Hypotension	13 (44.8)	10 (33.3)	0.262
Hypertension	0 (0)	5 (16.7)	0.008
Postoperative AE			
Nausea	1 (3.4)	3 (10.0)	0.612
Vomiting	0 (0)	1 (3.3)	1.000
Pulmonary infection	0 (0)	1 (3.3)	1.000
Atelectasis	1 (3.4)	1 (3.3)	1.000
Reoperation	0 (0)	1 (3.3)	1.000
Paresthesia	1 (3.4)	0 (0)	0.492

*AE* Adverse event, *PVB* Paravertebral block, *GA* General anesthesia. *All variables were analyzed using chi-square test

## Discussion

It is unknown whether thoracic PVB can be used in patients undergoing OPCABG. Therefore, this pilot study aimed to investigate the feasibility of bilateral PVB combined with GA in patients undergoing OPCABG. The results showed that nerve stimulator-guided bilateral thoracic PVB combined with GA could be efficient in OPCABG to provide high-quality analgesia. However, these findings should be interpreted with caution as non-anticoagulated patients were not assessed in this study. In addition, the risk of AEs is rather difficult to estimate, especially in case of small sample size.

Thoracic spinal nerve block has been clinically used since as early as 1905 and has become more popular in recent years due to a number of advantages: simple application, low failure rate, satisfactory analgesia, and less influence on respiration and circulation [26–28]. Previous studies reported unilateral thoracic nerve block combined with GA applied as analgesia in minimally invasive direct coronary artery bypass (MIDCAB) [29–31]. Satisfyingly, unilateral thoracic nerve block combined with GA exhibited remarkable efficacy in postoperative pain relief, while maintaining stable hemodynamics and less postoperative complications [29–31]. In this study, both rest and cough pain scores were lower in patients undergoing OPCABG and fewer patients received rescue analgesia within 24 h postoperatively in the PVB + GA group compared with the GA group. However, it should be noted that similar numbers of patients received rescue analgesia at 36 h and 48 h in both groups. These findings indicate some improvement in analgesia with PVB + GA compared with GA alone. Therefore, PVB may exert beneficial effects in patients undergoing OPCABG, which deserves further investigation.

Our study is the first comparative study to evaluate bilateral preoperative thoracic paravertebral block applied to patients undergoing OPCABG. We found that the thoracic nerve block segment was about 5 dermatomes in the PVB + GA group, which is in accordance with the imaging results from Christopher et al. [25] Our results also showed that a lower dosage of fentanyl was used during the operation and less PCA morphine was consumed at 24 and 48 h postoperatively. The extubation time and length of stay in the ICU were shorter in the PVB + GA group, but these factors have little clinical impact, if any.

As is well known, pneumothorax, puncturing of blood vessels, local hematoma, hypotension, and epidural block are common complications of thoracic PVB [32, 33]. Fortunately, no local anesthetic toxicity event occurred in the present study. The rate of AEs was not different in both groups. It is worth noting that the biggest PVS is close to the T_1–2_ or T_2–3_ intervertebral space, so it is safest to puncture at this site, as the risk for pneumothorax will be lower due to the greater distance between the parietal pleura and pyramis. That is also the reason why we chose T_3–4_ as the puncture point, which is close to T_1–2_ or T_2–3_ PVS, as it increased puncture reliability and reduced AE occurrence. In addition, anesthesia was terminated at 30 min before the end of the operation in this study, for the following reasons: 1) to achieve better postoperative analgesia; 2) to assess safety issues due to a high level of monitoring after cardiac surgery; and 3) since 15 ml of drug was injected on each side, PVB was prone to exert minimal impact on hemodynamics, ensuring stable circulation.

Hypotension is an AE that occurs in about 4% of pediatric patients [32]. In breast cancer surgery, PVB did not induce hypotension in any of the patients [34, 35]. Similar results were observed in healthy volunteers [36]. Nevertheless, PVB has a lower risk of hypotension than thoracic epidural analgesia [26, 37, 38]. In the present study, no hypotension occurred in the PVB + GA group, suggesting that hypotension is not a major risk in these patients. Nevertheless, this could vary among different populations of patients with different conditions. This warrants additional study.

In the present study, neuromuscular stimulation was used for PVB instead of the ultrasound-guided approach, which is now considered the best approach for PVB [39]. Nevertheless, neuromuscular stimulation is still a valid approach for PVB [22, 23, 40]. China is a developing country that is still adapting to modern approaches. Our hospital has just purchased the ultrasonography (USG) equipment, and the Anesthesiologist have not yet grasped the USG-guided technique. In addition, the learning curve of USG is steep [41]. Hence, even the nerve stimulator has been used in our hospital for many years, the nerve stimulator-guided technique was adopted in the present study until recently.

The present study is not without limitations. Despite the randomization, blinding, and control group, this was a pilot study with a small sample size from a single center. Since this was a pilot study, the sample size was estimated by referring to similar studies [26] rather than accurate calculation. In addition, our reduced sample does not allow any conclusions to be drawn about the safety of the procedure, whether for the morbidity of the technique or for the toxicity of local anesthetics. Our future multicenter randomized controlled trial will have a rigorous sample size calculation based on the results of the present study. Additional studies are necessary to confirm the effect of PVB for OPCABG.

## Conclusions

In conclusion, nerve stimulator-guided bilateral thoracic PVB combined with GA could be used efficiently in OPCABG with reduced rescue analgesia and morphine consumption. Additional studies are necessary to examine the potential AEs.

## Data Availability

All necessary data supporting our findings has been presented within the manuscript. The datasets used and/or analyzed during the current study are available from the corresponding author on reasonable request.

## Abbreviations

AE: Adverse event
ALT: Alanine transaminase
ANOVA: Analysis of variance
AST: Aspartate transaminase
BMI: Body mass index
BUN: Blood urea nitrogen
CI: Cardiac index
CPB: Cardiopulmonary bypass
CVP: Central venous pressure
ECG: Electrocardiogram
ETCO_2_: End-tidal carbon dioxide
GA: General anesthesia
HR: Heart rate
ICU: Intensive care unit
LVEF: Left ventricular ejection fraction
MAP: Mean arterial pressure
MIDCAB: Minimally invasive direct coronary artery bypass
MPAP: Mean pulmonary arterial pressure
OPCABG: Off-pump coronary artery bypass grafting
PCA: Patient-controlled analgesia
PCWP: Pulmonary capillary wedge pressure
PVB: Paravertebral block
PVRI: Pulmonary vascular resistance index
PVS: Paravertebral space
SBP: Systolic blood pressure
SCr: Serum creatinine
SD: Standard deviation
SpO_2_: Oxygen saturation
SVRI: Systemic vascular resistance index
TEA: Thoracic epidural anesthesia
USG: ultrasonography
VAS: Visual analogue scale
